# State of the Art in Stem Cell Research: Human Embryonic Stem Cells, Induced Pluripotent Stem Cells, and Transdifferentiation

**DOI:** 10.1155/2012/317632

**Published:** 2012-05-29

**Authors:** Giuseppe Maria de Peppo, Darja Marolt

**Affiliations:** The New York Stem Cell Foundation, 1995 Broadway, New York, NY 10032, USA

## Abstract

Stem cells divide by asymmetric division and display different degrees of potency, or ability to differentiate into various specialized cell types. Owing to their unique regenerative capacity, stem cells have generated great enthusiasm worldwide and represent an invaluable tool with unprecedented potential for biomedical research and therapeutic applications. Stem cells play a central role in the understanding of molecular mechanisms regulating tissue development and regeneration in normal and pathological conditions and open large possibilities for the discovery of innovative pharmaceuticals to treat the most devastating diseases of our time. Not least, their intrinsic characteristics allow the engineering of functional tissues for replacement therapies that promise to revolutionize the medical practice in the near future. In this paper, the authors present the characteristics of pluripotent stem cells and new developments of transdifferentiation technologies and explore some of the biomedical applications that this emerging technology is expected to empower.

## 1. Introduction

**Figure 1:**
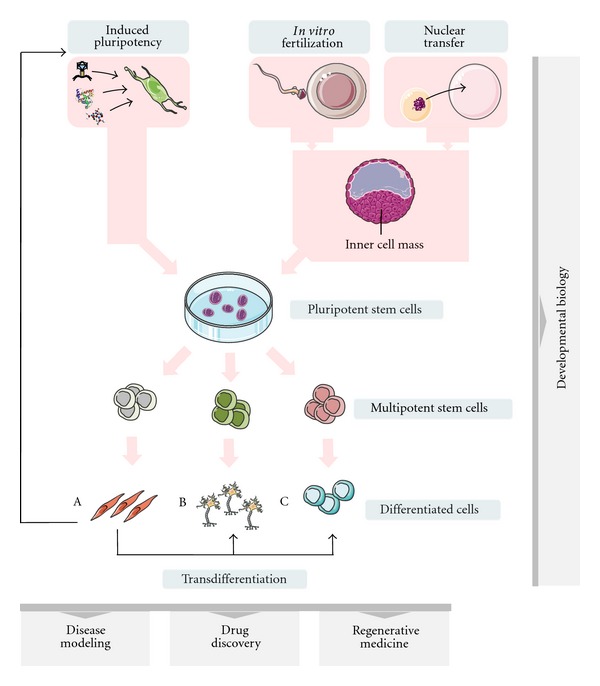
Human pluripotent stem cells and their biomedical applications. hESCs are isolated from early embryos obtained by *in vitro* fertilization or nuclear transfer, and give rise to more specialized cells (pink arrows). Alternatively, reprogramming technologies allow generation of hIPS from differentiated cells, or lineage conversion between differentiated cell types (black arrows). Developmental biology studies are unraveling the characteristics of cell types found at different stages. Stem cells and their differentiated progeny are used in a variety of biomedical applications, such as disease modeling, drug discovery, and regenerative medicine.

Stem cells represent the building blocks of our bodies, functioning as the natural units of embryonic generation during development, and adult regeneration following tissue damage [1]. They are defined by two distinct characteristics: the ability to maintain themselves through cell division, sometimes after long periods of inactivity (self-renewal), and the ability to give rise to more specialized cell types (differentiation) [2]. Based on the stage in development they are derived from, stem cells are broadly classified as embryonic, umbilical cord, and adult stem cells. Potency of stem cells decreases during development from totipotent stem cells at the morula stage, capable of differentiating into all embryonic and extraembryonic tissues, to pluripotent stem cells at the blastocyst stage, forming all embryonic tissues, and to multi- or uni-potent adult stem cells, forming tissues within their germ layer (Figure 1).

Modern understanding of stem cell biology dates back to 1960s, when multipotent hematopoietic and stromal stem cells residing in the bone marrow were identified [3]. From then on, a number of works have reported the isolation of stem cells from adult tissues. Adult stem cells, also called somatic stem cells, prompt tissue homeostasis throughout life and ensure tissue regeneration following damage. They reside in specific anatomic locations (“stem cell niches”) and are regulated by a combination of cellular, molecular, and physical signals [4]. Numerous types of adult stem cells have been identified, including hematopoietic, mesenchymal, endothelial, intestinal, and neuronal stem cells, each displaying different properties and potency. Mesenchymal stem cells, which have been isolated from a range of adult tissues including bone marrow [5], periosteum [6], fat [7], skeletal muscle [8], and synovial fluid [9], as well as from the cord blood [10], umbilical cord [11], and placenta [12], have been extensively explored as a source of cells for biomedical applications, owing to their broad regenerative potential, paracrine regulatory effects, and strong immunomodulatory activity [13]. Notably, cell populations isolated from different tissues exhibit phenotypic similarities, as well as specific differences in gene expression profiles and biosynthetic properties [14, 15]. Importantly, adult stem can be harvested and reimplanted in the same patient, therefore circumventing the immunological problems associated with allogeneic cell transplantation. However, adult stem cells exhibit limited proliferation potential and progressive loss of functionality upon *in vitro* expansion [16–18], and an age-associated decline in cellular fitness [19, 20], which limit their use for generating large amounts of functional cells for experimental and clinical applications.

On the other hand, human embryonic stem cells (hESCs), which have been first derived from early embryos in 1998 [21], are characterized by virtually unlimited proliferation potential and ability to give rise to all tissues constituting the human body, thus holding a high potential for biomedical research and clinical applications as an allogeneic cell source. However, the broader differentiation potential also presents a challenge in directing/controlling the cell fate, and a higher risk of tumor formation after transplantation of hESC-derived tissue progenitors compared to adult stem cells.

The ethical controversies involving isolation of hESC and the need for an autologous pluripotent stem cell source have fueled intense investigations into cellular reprogramming, leading to the generation of induced pluripotent stem cells (IPS) in 2006 [22] (Figure 1). More recently, the new techniques of cellular reprogramming have also been adapted for transdifferentiation—the ability of cells to switch from one specialized cell type to another directly, without reversal into a less specialized cell (Figure 1). We are now at a point when our growing understanding of the stem cell regenerative potential, and the development of new technologies offer unprecedented possibilities to conduct biomedical research and to translate the findings into new treatments. In the current paper, we will present the state of the art in human pluripotent stem cells and transdifferentiation research, and discuss some of the biomedical applications that this emerging technology is expected to empower.

## 2. Derivation, Culture, and Characteristics of**** Pluripotent Stem Cells

### 2.1. hESC Derivation and Culture

Thomson and colleagues were the first to derive hESC lines from the inner cell mass of human blastocysts generated during *in vitro* fertilization procedures [21]. Derivation from morula stage embryos, from developmentally arrested (poor quality) embryos, and from single blastomeres has also been reported, although with lower efficiencies [23–26]. Between 6–50% of normal embryos give rise to new hESC lines, whereas only up to 3% success has been reported with poor quality embryos [23]. Chen and colleagues systematically explored the timing of inner cell mass isolation and found that isolation on day 6 postfertilization results in the highest hESC derivation efficiency (~50%), about 10-fold increased compared to isolation on day 5, and about 5-fold increased compared to hESC derivation from intact blastocysts [27].

hESC typically grow in compact colonies on feeder layers of murine embryonic fibroblasts or human cells, which produce the extracellular matrix for cell attachment and condition the culture medium with paracrine factors [21, 28]. Protein substrates (e.g. matrigel, laminin, vitronectin) and synthetic matrices can also be used for cell derivation and/or culture, providing a more reproducible culture system [29, 30]. hESC culture media were initially supplemented with fetal bovine serum of selected lots, or serum replacement, and with growth factors (i.e., basic fibroblast growth factor) which activate the intracellular signaling networks maintaining pluripotency [21, 31]. In 2006, Ludwig and colleagues developed a defined medium for feeder-free derivation and culture of hESC [32], accomplishing one of the critical steps to standardize cell production and banking. However, hESC cultivation and passaging are conducted manually, using enzymes (collagenase IV, dispase, trypsin) or mechanical dissociation of colonies. Survival of hESC as single cells or small aggregates at passaging and after freezing-thawing cycles has been extremely low, until the discovery that application of a selective Rho-associated kinase (ROCK) inhibitor, Y-27632 markedly diminishes dissociation-induced apoptosis [33]. Further investigations are aimed at automating and scaling up hESC production, such as expansion and differentiation in three-dimensional carriers in suspension culture bioreactors [34].

hESCs are commonly characterized by probing the expression of pluripotency markers, including transcription factors (Oct4, Sox2, Nanog), surface antigens (stage specific antigens SSEA-4, SSEA-3, proteoglycans TRA-1-60, TRA-1-81), and enzymes (alkaline phosphatase and telomerase). Pluripotency is confirmed by testing the formation of teratomas containing tissues of all three germ layers after injection into immunocompromised mice [21, 35]. In addition, genetic status and microbiological status are screened to assess quality of the cultures. The described hESC culture environment has allowed extensive (potentially unlimited) expansion of undifferentiated cells, while maintaining a normal euploid karyotype. However, several studies showed that hESC cultivation can lead to accumulation of genomic abnormalities, including amplifications and deletions of whole chromosomes or large genomic regions, amplification of certain gene regions (i.e., MYC oncogene), changes in single nucleotide polymorphism, mutations in mitochondrial genome, and aberrant methylation profiles [36–38]. Many of these alterations were observed in late passages, and are likely to provide a growth advantage to the cells. In the future, it will be important to evaluate how specific genomic changes might affect the differentiated progeny of hESC, and to regularly monitor the genomic status of hESC applying high-resolution techniques.

### 2.2. hIPS Derivation, Culture, and Similarity to hESC

First indications that nuclei of somatic cells can be reprogrammed to generate all germ layers of an adult animal date back to 1958, when Gurdon and colleagues cloned mature fertile *Xenopus laevis* from cultured intestinal cells of tadpoles [39]. It took until 1997 to clone the first mammalian, Dolly the sheep, by transferring the nucleus of an epithelial cell into enucleated oocyte [40]. Reports of successful cloning of mice, cats, dogs, and other animals by somatic cell nuclear transfer followed [41–43]. Recently, it was shown that human somatic cell nuclei can be reprogrammed when intact oocytes are used as the recipient cells, resulting in the first triploid pluripotent cell lines [44]. Meanwhile, a groundbreaking discovery that has transformed the field came from an alternative approach in 2006/2007, when Takahashi and colleagues reprogrammed first mouse and then human skin fibroblasts to an “induced pluripotent” state by forced expression of four transcription factors: *Oct4*,* Sox2*, *Klf4*, *c-Myc*, delivered by retroviruses [22, 45]. Understanding of ESC biology was essential for the generation of induced pluripotent stem cells (IPS), guiding the selection of transcription factors and culture conditions. Improvements of the reprogramming methods followed rapidly, including the use of alternative nonintegrating vectors [46, 47], reprogramming by recombinant proteins [48], using only two factors for reprogramming [49], and replacement of transcription factors with small molecules [50]. In 2010, Warren and colleagues reported on cellular reprogramming using modified RNA, avoiding genetic manipulation, and increasing the efficiency of the process [51]. Whereas optimization of techniques is still ongoing, generation of hIPS lines in a safe way opens the possibility for their use in replacement therapies.

Many of the culture protocols developed for hESC have been successfully adapted to hIPS, which resemble hESC in their morphology, feeder dependence, surface marker expression, and *in vivo* teratoma formation capacity. However, it remains an open question how similar are various hESC and hIPS lines. A study by the International Stem Cell Initiative characterizing 59 hESC lines from 17 laboratories showed similarities in the expression patterns of most hESC markers, as well as differences in expression patterns of some lineage markers and imprinted genes [35]. Variable efficiencies to form specific lineages, such as cardiac, neuronal, and pancreatic *in vitro*, were also observed [52, 53]. In hIPS, the problem of variability resulting from different genetic backgrounds and culture conditions is compounded by the differences in reprogramming protocols, which involve major transcriptional and epigenetic shifts. Perhaps not surprisingly, studies showed larger heterogeneity of hIPS compared to hESC on a single-cell level [54], distinct gene expression and methylation signatures, epigenetic memory of somatic cell origin, and differences in the yield and properties of differentiated progeny, such as early senescence of IPS-derived cells [55]. There have also been reports of similarities between cohorts of hESC and hIPS, such at the study of Boulting and colleagues, who have found a similar range of efficiencies for motor neuron differentiation between hESC and hIPS lines [56]. Bock and colleagues have approached the problem by establishing genome-wide reference maps for DNA methylation and gene expression of 20 hESC and 12 hIPS lines, and combined these with assaying *in vitro* differentiation propensity and evaluating 500 lineage-specific transcripts. The assays were combined in a scorecard for a quick and comprehensive characterization of pluripotent cell lines [57]. Their results suggest that hESC and hIPS should be regarded as two partially overlapping groups, with inherent variability among both hESC and hIPS lines. Some studies have indicated that hIPS retain certain epigenetic memory from the parent cells [58], and it is largely unclear to what extent the origin of the reprogrammed cells could affect their safety and function. Ideally, the source cells would be easily accessible with minimal risk procedures for the patients (i.e., blood sample), available in large quantities, and allow relatively high reprogramming efficiency and fast hIPS derivation process.

## 3. Applications of Pluripotent Stem Cells

### 3.1. Pluripotent Stem Cells Offer New Insights into Developmental Biology

The elucidation of the molecular and cellular events occurring during embryogenesis is of paramount importance to understand the key pathways regulating cell differentiation and tissue formation under normal and pathological conditions. Historically, early development was studied in experimental animals, with mouse being the most popular due to its defined genetics and reproductive ability. Strong conservation of genes and signaling pathways regulating development between the species has justified the use of animal models as research platform [59]. Mouse studies led to identification of several genes and signaling pathways, including Wnt, Nodal and BMP pathways, which display specific spatiotemporal expression profiles during embryogenesis and play an important role in germ layer specification and tissue formation (reviewed by Tam and Loebel) [60]. Subsequent studies in mESC cultures revealed that the same pathways are involved in the regulation of germ layer development *in vitro* [61].

Despite the similarities observed, there are also differences between mouse and human development, including the timing and profile of expression of control genes, as well as the onset and patterning of tissue formation [62, 63]. In addition, discrepancies exist between mESC and hESC, which differ in morphology, cell surface markers, responsiveness to specific factors (including the leukemia inhibitory factor signaling pathway), and pluripotent state [64]. Noteworthy, recent studies demonstrated that hESC display a closer similarity with mouse epiblast stem cells (EpiSC), which are derived from postimplantation embryos [65], suggesting that mouse EpiSC might be a closer equivalent for comparative developmental studies between the two species.

Today, the ability to culture human pluripotent stem cells and to apply developmentally relevant inductive signals offers an unprecedented possibility to establish human models of embryonic development *in vitro*. Early lineage specification is recapitulated when ESC are induced to form three-dimensional cell aggregates embryoid bodies (EBs) [66]. In order to direct differentiation toward specific cell lineages, EBs are stimulated with specific growth factors and signaling molecules in tightly controlled concentrations and temporal sequences. Typical factors currently used to promote human EBs differentiation include EGF, FGF, RA, BMP-4 for ectodermal-mesodermal induction, TGF-*β* and activin-A for mesodermal induction, and a set of small molecules for endoderm specification [61, 67]. Examples of specific lineages derived from hESC using directed differentiation approach include functional motor neurons [68, 69], human cardiovascular progenitors [70], pancreatic endoderm [71], insulin-secreting *β*-cells [72], hepatocytes [73], and others. Importantly, cells exhibited functionality following *in vivo* implantation, such as improvement in cardiac function in rodent models of myocardial infarction [70] and development of glucose-responsive endocrine cells from implanted pancreatic endoderm [71]. Similarly, stepwise induction protocols have been successfully used for differentiation of IPS lines toward different specialized cell types, although the authors often report differences in differentiation efficiency and properties of differentiated progeny [48, 74, 75]. Factors such as size of EBs and variability of initial hESC colonies have also been shown to affect differentiation outcomes [76, 77]. For some lineages, differentiation has been achieved in monolayer culture [78], thereby simplifying the protocols and avoiding the variability associated with EB cultures. Frequently, a combination of EBs and monolayer culture is used [79], utilizing the effects of three-dimensional environment on cell differentiation, and accessibility of monolayer culture for subsequent cell characterization.

### 3.2. Disease Modeling and Drug Discovery Using Pluripotent Stem Cells

hESC and hIPS technologies allow the generation of novel *in vitro *models to study the underlying mechanisms of disease development, and establish platforms for the screening of new drugs to prevent or reverse disease progression. In one of the first examples, DiGiorgio and colleagues have shown that motor neurons derived from hESC are sensitive to toxic effects of glial cells that carry a mutation in SOD1 gene, causing amyotrophic lateral sclerosis (ALS), a fatal neurodegenerative disease [68]. To study the familial forms of the disease, the group has established hIPS lines from patients with ALS and shown they can be differentiated into motor neurons [74]. Development of models to study inherited and degenerative disorders, including Alzheimer's disease [80], Parkinson's disease [81], schizophrenia [82], diabetes [83], Gaucher's disease [84], and muscular dystrophy [84] is ongoing and expected to significantly affect our understanding of a variety of medical conditions. Creation of disease-specific hIPS is especially valuable when the underlying mechanisms of the disease are not well understood, and the affected cells are not available from the patients, for example in neurodegenerative diseases or cardiac conditions.

Generation of hESC/hIPS libraries from specific patient populations and healthy individuals also allows the development of screening platforms to evaluate toxicity and safety of existing and new drugs. During recent years, a considerable number of drugs were withdrawn from the market due to unforeseen cardiotoxic side effects, and cardiotoxicity is one of the major reasons for late-stage attrition of drug candidates. Another serious complication is drug-induced liver injury, which is among the most frequent reasons for withdrawal of approved drugs from the market. The early detection of fatal side effects of new drugs could prevent the continuation of a useless and cost-intensive developing process. Also, exclusion of compounds proving false negative in suboptimal test systems could be prevented by improving detection of potential toxicity. Currently, negative effects are not detected during *in vitro* and preclinical studies, due to the limited functional capacity and genetic diversity of cellular models used, and due to the interspecies differences between experimental animals and humans in pharmacotoxicological effects [85]. The potential usefulness of hESC-derived cardiac cells for safe pharmacology was shown recently [86, 87], where cells were reactive to several drugs in a manner that resembles mature cardiomyocytes. In addition, there has been progress in the establishment of protocols for the generation of hepatocyte-like cells from hESC and hIPS [85]. With respect to evaluating hepatotoxicity using these cells, it will be important to establish the degree of their similarity to primary cells, as well as to identify specific assays to evaluate the functionality of the cells and to detect the drug-induced liver injury *in vitro*.

### 3.3. Tissue Engineering and Cell Therapy Using Pluripotent Stem Cells

A large number of medical conditions are associated with loss or malfunction of tissues and organs, and result in patient distress, disability, and death [88]. Treatments for these patients currently rely on the transplantation from living and deceased donors, or implantation of medical devices that are limited in their functionality and availability. The ability of stem cells to self-renew and differentiate into specialized cells offers great possibilities in the field of regenerative medicine, both for the *ex vivo* construction of tissue substitutes (tissue engineering) and for transplantation of healthy, potentially genetically-corrected cells (cell therapy).

In tissue engineering, stem cells are combined with biomaterial scaffolds, which act as a functional template for regeneration and tissue maturation under appropriate culture conditions [88]. To date, adult stem cells have largely been used for experimental and clinical tissue engineering applications [89]. However, adult stem cells display limited proliferation and differentiation potential, progressive loss of functionality upon *in vitro* expansion, and age-associated decline in cellular fitness [16, 18–20, 90]. In contrast, pluripotent stem cells allow the generation of an unlimited supply of reparative cells (which are patient-matched in the case of hIPS). To avoid the risk of teratoma formation following *in vivo* implantation, hESC and hIPS are induced into lineage-specific progenitors, which display differentiation potential restricted within the germ layer [91, 92]. In one example, our group and others have derived mesodermal progenitors with similarity to adult mesenchymal stem cells in the expression of surface markers, global gene expression profile, and potential to differentiate into osteogenic, chondrogenic, and adipogenic lineage [91, 93–95]. hESC-mesenchymal progenitors have displayed excellent potential for formation of bone-like tissue when seeded in three-dimensional scaffolds and cultured in dynamic culture systems, suggesting they could be a promising cell source for regeneration of the skeletal system [95]. In some studies, *in vivo* transplantation of hESC-mesenchymal progenitors showed restricted developmental potential of the cells, as teratoma formation was not observed [94, 95]. In addition, hESC-mesenchymal progenitors injection improved the outcome of ischemic hindlimb injuries [94]. In light of the variability detected between hESC and hIPS lines, the current challenges lay in the development of robust protocols that will allow effective and reproducible differentiation of various hIPS lines. In addition, therapeutic applications of hESC and hIPS derivatives will critically depend on the development of stringent protocols for selection and/or purification of well-defined, stable tissue progenitors, that will demonstrate limited developmental potential within the selected lineage.

Advances in the hESC field have already resulted in first clinical trials using hESC-derived cells. In 2010, Geron Corporation started a phase I clinical trial to evaluate the safety of hESC-derived neural progenitor cells administration in patients with neurologically complete subacute spinal cord injuries (http://clinicaltrials.gov/, number NCT01217008). Unfortunately the trial has been stopped in 2011 due to financial difficulties of the company [96]. Currently, Advanced Cell Technology has two ongoing prospective clinical trials (phases I/II) to evaluate the effects of subretinal injection of hESC-derived retinal pigment epithelium cells in patients with dry age-related macular degeneration and patients with Stargardt's macular dystrophy (http://clinicaltrials.gov/, no. NCT01345006 and NCT01344993). Preliminary studies indicated measurable improvements in the vision of two nearly blind patients that lasted four months following the treatment [97]. A similar clinical trial for treatment of age-related macular degeneration with hESC-derived retinal pigment epithelium cells is expected to start in the UK in 2012 by The London Project To Cure Blindness (http://www.thelondonproject.org/).

In addition to tissue replacement, stem cells represent a valuable tool in cell therapy for the correction of genetic diseases. In one of the recent studies, Deyle et al. reported the disruption of the allele carrying the dominant mutation in the type I collagen genes causing osteogenesis imperfecta, using a combination of gene-targeting and IPS technology [98]. Mesenchymal stem cells derived from the bone of patients affected by the disease were gene targeted to disrupt the mutant alleles causing the disease, converted to hIPS, expanded *in vitro*, and then induced to differentiate toward the osteogenic lineage. Following *in vivo* implantation on ceramic scaffolds, gene-targeted osteogenic cells were able to form bone tissue in experimental animals [98]. An alternative approach—correction of genetic mutations was reported for other diseases, including *α*1-antitrypsin deficiency and X-linked chronic granulomatous disease, using the zinc finger nuclease technology, which holds great potential for efficient and precise gene manipulation at the desired genomic locations [99, 100]. In summary, stem cell technology, along with the accurate genetic manipulation of hIPS, opens new possibilities for the generation of clinically relevant cells for autologous cell-based therapies.

## 4. Transdifferentiation

Our growing understanding of the stem cell biology and the development of specific cell lineages has allowed the generation of multiple specialized cell types from stem cells, and the identification of distinct developmental stages that are governed by genetic and epigenetic regulatory networks. In parallel, an alternative approach is being investigated, in which one somatic cell type could be directly converted—transdifferentiated—into another somatic cell type. This way, abundant adult cells, such as dermal fibroblasts or adipocytes, could be used to produce other therapeutically important cells, such as neurons, cardiomyocytes or pancreatic beta cells [101]. Natural transdifferentiation involves a stepwise dedifferentiation of the primary cell to an intermediate cell type, that can then differentiate into the new lineage. In contrast, experimentally induced transdifferentiation involves a direct conversion through a simultaneous downregulation of one genetic programme and a concomitant upregulation of the new genetic programme [102].

In one of the first experiments, muscle-specific genes were induced in pigmented epithelium, nerve, fat, liver, and fibroblast cells by forced expression of MyoD, a master regulator of muscle differentiation [103]. However, it was not clear until 2008 how the procedure could be applied more generally to other cell types. Following the IPS reprogramming experiments, Zhou and colleagues found that reexpressing a combination of 3 transcription factors, tested from a list of 20 key developmental regulators, can turn pancreatic exocrine cells into pancreatic beta-like cells in adult mice [104]. Using a similar approach, Vierbuchen and colleagues showed that a combination of 3 factors with important roles in neural development converts mouse embryonic and postnatal fibroblasts into functional neurons *in vitro* [105]. In a later study, it was shown that the same factors work in converting terminally differentiated hepatocytes into neurons, thereby demonstrating the possibility to reprogram cells between different germ layers [106]. Interestingly, the resulting neurons silenced the donor cell transcriptional program, suggesting a binary lineage switch rather than an induction of hybrid cell phenotypes. However, a small but detectable epigenetic memory of the donor cells remained in the induced neurons [106].

Current examples of experimentally induced transdifferentiation rely on the ectopic expression of master regulator genes, which are essential for lineage specification during development and can direct the establishment of a new epigenetic state when expressed in the host cell. To achieve a complete phenotypic switch between the two mature cells and generate functional cells for potential clinical applications, the activation of a new epigenetic state in the host cells must be associated with full deactivation of the original epigenetic state. This might be more cumbersome to achieve between more distantly related cells owing to their larger epigenetic differences, resulting in relatively inaccessible chromosomal regions [101]. Possible strategies to convert more distant cell types could involve the use of chemicals that loosen the chromatin structure and favor epigenetic rearrangements [107]. In addition, reduction of factors that support the original epigenetic state of the mature host cells, and the use of factors that promote the reentry into cell cycle could facilitate the transdifferentiation [101].

Taken together, transdifferentiation studies underline the importance of specific factors in lineage specification, and overturn the long-believed notion of stable and irreversible lineage commitment events. With further development and optimization, transdifferentiation protocols could offer a shortcut to production of differentiated cells for biomedical research and clinical applications, potentially avoiding the extensive cell proliferation required for hIPS derivation and differentiation. In addition, transdifferentiation opens the possibility to convert different cell types directly *in vivo* to drive tissue repair and regeneration *in situ*, an approach that would be impossible using IPS technology due to the teratoma-forming ability of pluripotent stem cells. On the other hand, direct conversion between terminally differentiated cells, which are characterized by limited proliferative potential, may not be suitable for experimental and clinical applications where large amounts of cells are needed, and in situations where disease conditions result from genetic mutations. Hitherto, only a few instructive factor combinations have been reported to directly convert mature cell types, and it is likely that these will depend on the selected target and final cell types. As with the IPS, a number of questions regarding the transdifferentiated cell stability, functionality, and safety need to be addressed, and a deeper understanding of the reprogramming process is needed before transdifferentiation can be translated into a therapeutic setting.

## 5. Conclusions

Advances in pluripotent stem cell biology and transdifferentiation have generated great enthusiasm in the scientific community over the last years, and are expected to affect numerous biomedical applications. hESC and hIPS allow the study and modeling of human development and tissue formation under diverse experimental conditions, and therefore represent an excellent platform for understanding human diseases and developing innovative therapeutic solutions. In parallel, development of transdifferentiation technology offers new insights into adult cell plasticity and could lead to expedited protocols for differentiated cell production. In the context of regenerative medicine, hESC and hIPS offer the possibility to generate an unlimited number of functional cells for reconstructive and reparative therapies. Creation of hIPS libraries is underway, and will expedite both safety pharmacology studies, as well as development of novel therapies. Approval of the first hESC-based clinical trials in the USA is encouraging for the field, and signifies the potential of pluripotent stem cells to cure the most devastating diseases of our time. However, there is a lot of work to be done to fully determine the clinical potential, efficacy, and safety of hESC and hIPS-based treatments. As with other medical breakthroughs, it is only via scrupulous scientific investigation that the true potential of human pluripotent stem cells may be realized.
